# The gender gap in sickness absence from work and the influence of parental absence on offspring absence 15 years later: register-based cohort of Norwegians born in 1974–1976

**DOI:** 10.1186/s12889-015-2037-2

**Published:** 2015-07-21

**Authors:** Petter Kristensen, Karina Corbett, Ingrid Sivesind Mehlum

**Affiliations:** Department of Occupational Medicine and Epidemiology, National Institute of Occupational Health, Oslo, Norway; Department of Community Medicine, Institute of Health and Society, University of Oslo, Oslo, Norway

## Abstract

**Background:**

Women have shown consistently higher levels of sickness absence from work in comparison to men, but explanations for this gender gap have not been completely understood. Life-course studies suggest that health and health-related social benefits in adult age are influenced by early life experiences. We aimed to estimate intergenerational associations with a 15-year time gap between parents’ and offspring sickness absences, pursuing the hypothesis that this parental influence would have a stronger impact for women than for men.

**Methods:**

All persons born alive between 1974 and 1976 in Norway were followed up in several national registries. Employed persons considered to be at risk of sickness absence and also with parents at risk of sickness absence (*n* = 78 878) were followed in the calendar year of their 33^rd^ birthday with respect to spells lasting >16 days. The probability of one or more spells during this year constituted the one-year risk under study. Additive risk differences in association with an exposure (parental sickness absence 15 years earlier) were estimated in a binomial regression analysis. The estimates were adjusted for parental socioeconomic factors.

**Results:**

The 1-year sickness absence risk was higher for women (30.4 %) than for men (12.3 %). The crude risk differences between those exposed and those unexposed to parental sickness absence were similar in percentage points (PP) for women (3.8; 95 % confidence interval (CI) 2.6 to 4.9) and men (3.8; 95 % CI 2.9 to 4.6). The risk differences were moderately attenuated after adjustment for parental education and father’s income to 3.4 PP (2.2 to 4.5) for women and 2.8 PP (2.0 to 3.7) for men. Male absence was more strongly associated with the father’s than with the mother’s sickness absence, while associations for women were stronger for the same diagnostic groups as their parents.

**Conclusions:**

Parental sickness absence was moderately associated with sickness absence in the next generation. Bias from unmeasured confounders cannot be entirely dismissed. Contrary to our hypothesis, associations were not stronger for women than for men. If parental sickness absence has a long-term causal effect, preventive measures could have an impact over generations.

**Electronic supplementary material:**

The online version of this article (doi:10.1186/s12889-015-2037-2) contains supplementary material, which is available to authorized users.

## Background

Determinants of sickness absence are extensively studied internationally, and reported risk levels have consistently been higher for women than for men [1–8]. Explanations for this gender gap have been scrutinized but are not fully understood [1–3, 7]. The gap could be to some degree due to gender inequalities either at home (the double burden) [4, 9–11] or at work [3, 4, 6, 12–15]. Biological differences between females and males, notably matters related to pregnancy and birth, explain a considerable part of the absence gap [5, 10, 16, 17]. Part of the gender gap could also be due to a higher female morbidity not restricted to pregnancy and birth [6]. Furthermore, women could have a lower threshold or a higher susceptibility for perceiving health problems than men [17, 18].

Social interaction is a concept in econometrics [19] that has been introduced as a potential cause of different outcomes, including sickness absence. This concept means that own risk of sickness absence could be influenced by the level of sickness absence in the surroundings among colleagues [20, 21], neighbors [22, 23], and family members [22]. However, it has not been clearly established if either sex is more susceptible to these proximate exposures [20–22]. So far, explanatory models have usually been dominated by contemporary factors [1]. Therefore, there is a research gap concerning mechanisms for sickness absence that act over long time spans and across generations. The life course concept could, in this respect, offer an alternative and a new opportunity to understand adult age behavior and health [24–26]. Studies of life course cohorts suggest that sickness absence can be influenced by social conditions in early life [24, 26] and that there are differences between diagnostic subgroups of absence [5, 26, 27]. It is also worth noticing that the risk of permanent medical disability pensioning is related to parents’ disability [28–30]. Additionally, transmission over generations could be more directly related to disease and health perception [31, 32]. This has particularly been observed for depression and other mental disorders [32–34]. Finally, twin studies suggest that long-term sickness absence could be transmitted over generations through genetic factors [35].

The present study was set up to investigate whether the gender gap in sickness absence for individuals born in Norway between 1974 and 1976 could be explained in part as an effect over generations. We have established a cohort based on data collected in national registries, including repeated measures of social circumstances and health for cohort members and their parents [26]. This gave an opportunity to estimate associations between parental and own sickness absence risk. Our study hypotheses were that parental sickness absence has an impact on own absence risk 15 years later and furthermore, that women are more susceptible than men to this parental influence. A supplementary aim was to explore whether the patterning of these relationships could shed light on the mechanisms behind the associations. We expected that causal effects would be stronger for sickness absence exposure from a parent of the same sex compared with the parent of opposite sex, and stronger for similar diagnostic categories compared with dissimilar diagnostic categories between parent and offspring.

## Methods

### Participants and data collection

The source population included all 169 498 live births listed in the Medical Birth Registry of Norway (MBRN) between 1974 and 1976. The unique national identification numbers of these persons and their parents allowed the linkage of national registries. Statistics Norway’s events database FD-Trygd [36] compiles register data from several sources, such as the sickness absence registry of the Norwegian Labour and Welfare Administration and the National Population Register. Educational data are registered in the National Education Database [37]. Permission to use MBRN data was given by the Norwegian Public Health Institute, Statistics Norway gave permission to use data from the FD-Trygd database and the National Education Database, and the use of sickness absence diagnoses was permitted by the Norwegian Labour and Welfare Administration. The Norwegian Tax Administration permitted to use data from the National Population Register. The Regional Committee for Medical Research Ethics approved the study (reference number S-06028a).

**Figure 1:**
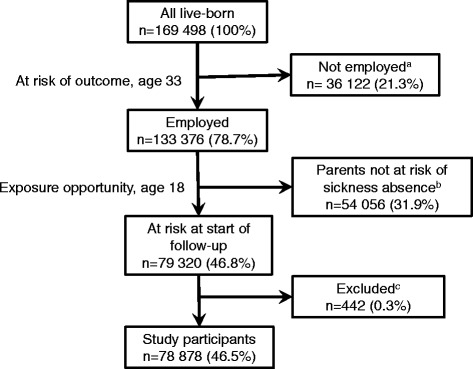
Flow diagram of live-born persons in Norway, 1974–1976. ^a^ Persons who died (*n* = 4330; 2.6 %) or emigrated (*n* = 11 826; 7.0 %) before age 33 years, or were not employed at age 33 years (*n* = 19 966; 11.8 %). ^b^ Excluded because one or both parents were not considered to be at risk of sickness absence at index person age 18 years (1992, 1993, or 1994). In categories that were not mutually exclusive: 21.5 % (*n* = 28 692) were excluded because their mother either received disability pension (*n* = 8585; 6.4 %), emigrated (*n* = 269; 0.2 %), was deceased (*n* = 1478; 1.1 %), was a government employee (*n* = 9252; 6.9 %), or had no income (*n* = 16 119; 12.1 %). A total of 26.8 % (*n* = 35 744) were excluded because their father either was not identified in the Medical Birth Registry of Norway (*n* = 12 514; 9.4 %), received disability pension (*n* = 6282; 4.7 %), emigrated (*n* = 269; 0.2 %), was deceased (*n* = 3376; 2.5 %), was a government employee (*n* = 11 107; 8.3 %), or had no income (*n* = 9325; 7.0 %). ^c^ Excluded because they received disability pension, emigrated, or died during the year of follow-up (2007, 2008, or 2009; age 33 years)

The study population encompassed persons (index persons) who, along with their parents, were considered to be at risk of sickness absence. A total of 133 376 index persons had registered employment in the calendar year of their 33^rd^ birthday (2007, 2008, or 2009). These index persons were included if both parents were identifiable in the MBRN, were national residents, had pensionable incomes, and were not disability pensioners at index person age 18 years. Index persons with at least one parent employed by the government in the same year were excluded because government employees were not included in the national sickness absence insurance scheme before 2000. Altogether, 54 056 individuals (31.9 % of the total) were excluded because at least one parent did not fulfill the criteria. An additional 442 individuals (0.3 %) were excluded because they received disability pension, died, or emigrated during the year of follow-up. The remaining 78 878 persons constituted the participants. A more detailed outline of the establishment of the study population is provided in Fig. 1.

### Study outcome (index person sickness absence)

The study outcome was the occurrence of any or diagnosis-specific sickness absence for the index person during the calendar year of the 33^rd^ birthday. The terms “sickness absence” and “absence” are used interchangeable for the outcome in this paper. Disability pension is a separate benefit in Norway and was not included. Information regarding spells lasting more than 16 days was retrieved from FD-Trygd. Employees in Norwegian enterprises are fully paid by the employer during certified sickness absence. The employer is reimbursed by the Labour and Welfare Administration after absence for 16 days absence, and registration is, therefore, considered to be complete for employees. Spells are detailed in the registry, including the dates of start and termination and diagnosis. The diagnosis is provided by the patient’s physician, who is obliged to label all sickness absence forms with an International Classification of Primary Care (ICPC) diagnostic code [38]. Spells occurring during the calendar year of the participant’s 33^rd^ birthday were included. All persons with sickness absence that fulfilled the duration criterion were classified in dichotomous ever-never categories: all-cause absence, pregnancy-related diagnoses (ICPC W), musculoskeletal diagnoses (ICPC L), psychiatric diagnoses (ICPC P), and all with absence(s) who did not receive a registered diagnosis during follow-up. Back disorders (ICPC L02, L03, L84, L86) and depression (ICPC P03, P76) were also considered. We further identified women with sickness absence(s) other than pregnancy-related as a specific outcome category.

Participant, not absence spell, was the unit of observation, and each person could, therefore, contribute to several diagnostic categories.

### Exposure (parental sickness absence)

Any parental absence spell during the calendar year of the index person’s 18^th^ birthday (none/any) served as the exposure variable (1992 for those born in 1974, 1993 for those born in 1975, and 1994 for those born in 1976). Several exposure subgroups were considered: parent-specific sickness absence (i.e., mother, father, none, both) as well as the same diagnostic groups and subgroups as those of the index person.

The same criteria were applied for classifying parental and index person sickness absence; however, the ICPC classification system was introduced in the early 1990s, and it was only partly in use in 1992. A diagnostic classification system based upon a 61-item code was used by the Labour and Welfare Administration prior to and parallel with the ICPC system. If the parental ICPC code was missing this other code was used.

### Covariates

The MBRN included data on year of birth, birth order, and both parents’ age and identification numbers.

Data on several parental variables in the year the index person turned 18 years were retrieved from FD-Trygd [36]: marital status (mother only), number and age of children in the household, date of death or emigration, geographical region of residence, governmental employment, and pensionable income. Income is recorded in basic units that are adjusted annually to be standardized to the general level of living. We divided each parent’s income into quartiles. FD-Trygd also included the date of death or emigration for the index person up to age 33 years.

Parental educational data at index person age 16 years were based on the Norwegian standard NUS2000 [37]. The nine-level educational attainment code was collapsed into five levels for each parent.

Details of covariate categorizations as applied in the analyses, their prevalence, and their distributions across parental and index person sickness absence are provided in Table 1.

**Table 1:** Distribution of the population characteristics, including exposure prevalence and risk of outcome

Characteristic	n	%	Exposure prevalence^a^	Outcome risk (%)^b^
Total	78 878	100	0.222	21.1
Sex				
Women	38 543	48.9	0.223	30.4
Men	40 335	51.1	0.222	12.3
Parental sickness absence				
Neither	61 331	77.8	0	20.3
Mother only	12 623	16.0	1	23.1
Father only	3746	4.7	1	24.9
Both	1178	1.5	1	31.7
Mother’s education level at index person age 16 years				
Tertiary, higher	971	1.2	0.120	16.4
Tertiary, lower	14 782	18.7	0.183	18.4
Upper secondary, complete	7755	9.8	0.174	19.3
Upper secondary, basic	38 205	48.4	0.223	21.7
Lower secondary or less	16 550	21.0	0.284	23.6
Missing	615	0.8	0.246	19.2
Father’s education level at index person age 16 years				
Tertiary, higher	6110	7.8	0.147	17.1
Tertiary, lower	13 775	17.5	0.172	18.7
Upper secondary, complete	17 276	21.9	0.213	20.7
Upper secondary, basic	25 836	32.7	0.239	21.8
Lower secondary or less	15 316	19.4	0.279	24.3
Missing	565	0.7	0.264	21.1
Father’s income quartile at index age 18 years				
1 (low)	19 693	25.0	0.273	22.6
2	19 201	24.3	0.254	21.8
3	20 313	25.8	0.201	21.2
4 (high)	19 671	24.9	0.163	19.0

^a^Parental sickness absence at index person age 18 years (any versus none)

^b^Index person sickness absence at age 33 years (any versus none)

**Figure 2:**
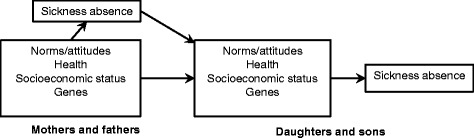
Causal diagram illustrating the relationship between parental and offspring sickness absence

A causal diagram illustrating the assumed relationship between parental and index person sickness absence is shown in Fig. 2. In this diagram, measured or unmeasured parental factors (i.e., norms and attitudes, health, socioeconomic position, and genes) could act as confounders whereas the same factors in the offspring generation could act as mediators between the parental factors and offspring sickness absence.

### Data analysis and statistics

We used Stata/SE 13.1 software (Stata Corporation, College Station, Texas, U.S.A). Participants were followed throughout the calendar year of their 33^rd^ birthday (2007, 2008, or 2009). The proportion with any absence or any diagnostic subgroup absence could therefore be viewed as a 1-year risk.

We estimated differences in sick leave risk between exposed and non-exposed participants using additive binominal regression with Stata’s *binreg* command. The additive risk differences are presented as percentage points (PP) and were analyzed separately for women and men. Ninety-five percent confidence intervals (CI) were computed for all associations. We were interested in adjusted risk differences but also crude estimates, as they represent the sex difference that was actually observed. Year of birth, birth order, region of residence, number of younger siblings in the household, and parental age, marital status, education and income were considered as potential confounders, as they commonly influence exposure and outcome. Only factors that changed the association between parental and index person absence by more than 5 % were determined to be actual confounders and included in the multivariable models. Factors related to parental socioeconomic position (the mother’s education, the father’s education, and the father’s income) fulfilled this criterion.

In addition to these main analyses, we conducted supplementary analyses relating to two questions that could elucidate the mechanisms behind the associations between parental and index person sickness absence:

- First, was the sex-specific association between parental and index person absence dependent on the sex of the parent with absence?
- Second, were the associations between parental and index person absence stronger for similar diagnostic categories compared to dissimilar diagnostic categories?

Additive binomial regression fails to converge if any estimate falls outside the 0 to 1 range. We had to exclude men with missing data on mother’s education or omit mother’s education from the model in some of the diagnostic subgroup analyses to obtain convergence.

Differences in adjusted exposure-outcome associations between women and men were computed by simple subtraction, with confidence intervals being computed as outlined by Altman and Bland [39].

We also performed sensitivity analyses to assess whether results were dependent on the *a-priori* choices in the participation criteria. This was performed by running analyses for all 133 376 subjects with registered employment at age 33. Parental sickness absence at index person age 18 years could be viewed as a misclassification problem if being exposed at a younger age would have been more relevant. We were able to assess this for index persons born in 1976 by comparing exposure at age 16 and age 18 (parental sickness absence 1992 vs. 1994). In addition to these attempts to assess selection bias and information bias, we assessed confounding by exploring the characteristics needed for unmeasured confounders to fully account for the observed exposure-outcome association. Here, we applied bias formulas according to VanderWeele and Arah [40].

## Results

Among the 78 878 participants, 16 671 (21.1 %) had a total of 21 531 sickness absence spells. Crude risks according to the covariate categories are shown in Table 1. Women had a higher all-cause risk (30.4 %) than men (12.3 %), even after disregarding pregnancy-related diagnoses that comprised approximately one-third of spells among women (Table 2). They had also higher absence risks than men in all diagnostic categories (Table 2).

**Table 2:** Diagnosis- and sex-specific sickness absence risk (%) among the study participants

Sickness absence category	All n (%)	Women n (%)	Men n (%)
No sickness absence	62 209 (78.9)	26 837 (69.6)	35 372 (87.7)
All-cause absence	16 671 (21.1)	11 706 (30.4)	4965 (12.3)
Musculoskeletal (ICPC L)^a^	5064 (6.4)	2798 (7.3)	2266 (5.6)
Psychiatric (ICPC P)^a^	3653 (4.6)	2429 (6.3)	1224 (3.0)
Pregnancy-related (ICPC W)^a^	3861 (4.9)	3859 (10.0)	2 (0.0)
Absence but no diagnosis	389 (0.5)	269 (0.7)	120 (0.3)
Other than pregnancy-related^a^		8092 (21.0)	

*ICPC* International Classification of Primary Care, 2^nd^ Edition

^a^Categories not mutually exclusive

The crude association between all-cause sickness absence and parental sickness absence was 3.8 PP for women (Table 3). This was mainly due to associations in the musculoskeletal and psychiatric categories. In contrast, pregnancy-related absence was not positively associated with parental absence. When adjusting for parental education and father’s income, the estimates were moderately attenuated, 26.7 % and 15.1 % for musculoskeletal and psychiatric diagnoses, respectively.

**Table 3:** Associations between parental sickness absence (exposure) and diagnosis-specific sickness absence among index persons: women

Sickness absence category	Absence risk	Crude risk difference	(95 % CI)	Adjusted risk difference^a^	(95 % CI)
All-cause absence					
Not exposed	29.5	0	Reference	0	Reference
Exposed	33.3	+3.8	(+2.6 to +4.9)	+3.4	(+2.2 to +4.5)
Musculoskeletal (ICPC L)					
Not exposed	6.7	0	Reference	0	Reference
Exposed	9.2	+2.5	(+1.8 to +3.2)	+1.8	(+1.2 to +2.5)
Psychiatric (ICPC P)					
Not exposed	6.0	0	Reference	0	Reference
Exposed	7.4	+1.4	(+0.7 to +2.0)	+1.2	(+0.5 to +1.8)
Pregnancy-related (ICPC W)					
Not exposed	10.2	0	Reference	0	Reference
Exposed	9.4	−0.8	(−1.5 to −0.1)	−0.4	(−1.1 to +0.3)
Other than pregnancy-related					
Not exposed	20.0	0	Reference	0	Reference
Exposed	24.5	+4.6	(+3.5 to +5.6)	+3.6	(+2.6 to +4.6)

*CI* confidence interval, *ICPC* International Classification of Primary Care, 2^nd^ Edition

^a^In a model including parental sickness absence, mother’s and father’s education level, and father’s income

Men had a pattern of associations that was quite similar to that of the women, for all-cause absence, musculoskeletal diagnoses, as well as psychiatric diagnoses (Table 4).

**Table 4:** Associations between parental sickness absence (exposure) and diagnosis-specific sickness absence among index persons: men

Sickness absence category	Absence risk	Crude risk difference	(95 % CI)	Adjusted risk difference^a^	(95 % CI)
All-cause absence					
Not exposed	11.5	0	Reference	0	Reference
Exposed	15.2	+3.8	(+2.9 to +4.6)	+2.8	(+2.0 to +3.7)
Musculoskeletal (ICPC L)					
Not exposed	5.1	0	Reference	0	Reference
Exposed	7.3	+2.2	(+1.6 to +2.8)	+1.6	(+1.0 to +2.1)
Psychiatric (ICPC) ^b^					
Not exposed	2.8	0	Reference	0	Reference
Exposed	3.9	+1.1	(+0.6 to +1.5)	+0.9	(+0.5 to +1.3)

*CI* confidence interval, *ICPC* International Classification of Primary Care, 2^nd^ Edition

^a^In a model including parental sickness absence, mother’s and father’s education level, and father’s income

^b^Three hundred eighteen men with missing information on the mother’s education level were excluded from the analysis of psychiatric diagnoses

The crude differences in risk differences between women as estimated in Table 3, and men as estimated in Table 4, were small: 0.0 PP (95 % CI −1.4 to +1.4) for all-cause absence; 0.3 PP (−0.3 to +1.0) for musculoskeletal diagnoses (largest for women); and 0.3 PP (−0.5 to +1.0) for psychiatric diagnoses (largest for women). Women had slightly stronger adjusted exposure-outcome associations than men, but these sex differences were clearly non-significant: 0.5 PP (95 % CI −0.9 to +1.9) for all-cause absence; 0.3 PP (−0.6 to +1.2) for musculoskeletal diagnoses; and 0.2 PP (−0.5 to +1.0) for psychiatric diagnoses.

When separating maternal and paternal exposure, we found that women’s absences, both all-cause and those within the diagnostic subcategories, were more strongly associated with maternal than paternal absence. These differences were however small and non-significant (see Additional file 1: Table S1). This pattern was clearer for men whose absences were more strongly associated with the father’s absence than with the mother’s absence, the difference being 2.3 PP (95 % CI +0.5 to +4.2) for all-cause sickness absence.

Both women and men showed stronger associations between own musculoskeletal absence and parental absence in the same diagnostic category compared with dissimilar parental diagnoses (see Additional file 1: Table S2). The same pattern was present for own psychiatric (ICPC P) absence and similar parental diagnoses. There were sex differences, however: women showed stronger and significant associations, as opposed to weaker and non-significant associations for men. Stronger associations for women were also evident with more specified diagnoses (back disorders, depression) being applied in both generations.

The sensitivity analyses showed that less restrictive participation criteria, including all 133 376 individuals with employment at age 33, reduced the risk difference estimates moderately but had no influence on the general pattern of similar additive differences in the exposure associations across genders (data not shown). Among the participants born in 1976, the associations between total parental and offspring all-cause sickness absence were stronger for exposure at age 16 years (parental absence in 1992) than exposure at age 18 years (parental absence in 1994). For women, exposure at age 16 years was associated with a risk increase of 4.9 PP (95 % CI 2.8 to 7.0) whereas exposure at age 18 years was associated with a risk increase of 2.9 PP (0.9 to 4.9). The corresponding estimates for men were 3.2 (1.7 to 4.8) and 2.2 (0.7 to 3.6), respectively.

We chose strong simplifying assumptions and hypothetical choices for the effects of unmeasured confounders on the exposure–outcome associations in the sensitivity analysis aimed at assessing the systematic error in risk differences due to unmeasured confounding. The resulting biasing effect is presented in the Appendix (see Additional file 1: Appendix). An unmeasured confounder associated with a 2.7-fold risk increase in parental absence, a 50 % risk increase in daughters’ absence, and a doubled risk increase in sons’ absence could have fully explained the observed associations between parental and offspring sickness absence.

## Discussion

Sickness absence at age 33 years was associated with parental sickness absence 15 years earlier among women and men born in Norway between 1974 and 1976. This was the case for musculoskeletal and psychiatric but not pregnancy-related diagnoses. The associations were only moderately attenuated by indicators of parental socioeconomic position. Contrary to the study hypothesis, these additive scale associations were of the same magnitude for women and men. Therefore, the results do not support the notion that intergenerational patterns between parents and offspring contribute to the gender gap in sickness absence.

### Strengths and limitations

This study was based on the linkage between national registries and contains repeated measures recorded throughout life. Linkage to parents and across registries is feasible because of the unique national identification number. The registries have complete nationwide coverage and missing information is generally a small problem. It is a virtue that the register data were included prospectively and that parent and index person absence data were collected independently of each other. These features lend considerable strength to the study.

One important problem relates to the selection of the study population. Norwegian sickness absence regulations are complex, being dependent on employment status, income level, and granting of other social benefits. Thus, the criteria for receiving sickness absence benefits in both generations could change during follow-up in a fashion that we were not able to track. Furthermore, those absent at the start of the follow-up were not excluded. Therefore, the proportion of index persons classified as absent should only be viewed as an approximation of a 1-year risk. However, the minor changes obtained in the sensitivity analysis of all employed persons render some confidence that the participation criteria had little influence on the results.

Limitations in data availability and quality were another potential source of bias. Register data are primarily collected for purposes other than research and are restricted in time according to the establishment of the registry in question as well as the time lag for the availability of the most recent data. Ideally, we would rather have had data on parental sickness absence at an earlier date than index person age 18 years, but we had to accept a trade-off because parental absence data were less reliable before 1992. Exposure information in an inappropriate time window could be viewed as a misclassification problem, which could result in an information bias toward the null. The sensitivity analysis of those born in 1976 showed stronger associations for exposure to parental sickness absence at age 16 years compared to age 18 years. This supports the assumption of misclassification and attenuation of exposure-outcome associations; however, exposure data were collected independently of outcome data, which would plausibly yield non-differential misclassification and tend to attenuate true associations rather than create false ones.

The most important validity problem was likely to be confounding. Parental socioeconomic position attenuated the intergenerational sickness absence association but only moderately. Adjustment could however be incomplete if socioeconomic factors that were not included (e.g., parental occupation) had additional influence or if the factors assessed were measured with error. Another, possibly more serious problem, was unmeasured factors that could be important confounders (Fig. 2). This is probably true for parental norms and attitudes, parental health, and genetic factors. Parental norms and attitudes are likely to fulfill criteria as confounders because they are determinants of parental sickness absence and also are able to influence sickness absence-prone norms and attitudes in their offspring. However, the sensitivity analysis suggests that such confounding would need to be strong to fully explain the results, particularly for men (see Additional file 1: Appendix). Indeed, the simplifying assumptions made in this analysis were strong, and caution of inferences from this finding is warranted.

### Comparison with other studies and inferences

The associations between parental and offspring sickness absence in this study are supportive of effects of social interaction and parental influences and in agreement with several econometric studies [20–23]. One consistent feature of social interaction is that the influence of close peer groups is stronger than that from more distant groups [22]. We found that men were more influenced by their fathers than mothers. This could favor a causal explanation or could also be due to confounding if the father’s sickness absence did not matter in itself but rather was a proxy for paternal norms and attitudes. The econometric literature addresses different effects of peer behavior, including sickness absence, that are interpreted as causal [19]. Study designs include natural experiments [21] and fixed effects analysis [22] to effectively control for unmeasured confounding. In an investigation of the Norwegian working age population, the fraction receiving social benefits (sickness absence or others) was slightly increased if near family members (i.e., parents, siblings) had received social benefits the preceding year [22]. One main difference between most econometric literature and our study is that econometric studies usually are set out to estimate the proximate effects of intervention policy measures, whereas we investigated relationships with a 15-year lag.

We are not aware of other studies addressing the long-latency relationships of sickness absence over generations. However, studies of other outcomes indicate that such effects exist. Permanent disability pensioning level seems to be dependent on parental disability [28–30]. Medically unexplained symptoms among 36-year old participants of the 1946 British birth cohort were associated with parental health status and the father’s long-term sick leave 21 years earlier [31]. Depression and other mental health problems in adult age were reported to be more common for persons growing up with parents with similar health problems [32–34]. Women seemed to be more susceptible than men to the influence of parental depression in one study [33], which could be in agreement with our finding of a stronger female association with parental absence in the same diagnostic groups.

We found no support for the study hypothesis of stronger associations for women than for men. This finding was unexpected and is not in accord with the assumptions of general female susceptibility with regard to sickness absence [3]. The results concerning gender differences in econometric studies are more inconsistent [20–22].

It is not easy to identify a causal effect of parental absence in this observational study where several parental factors, which are likely to be confounders, were not measured. One possibility is a causal effect of parental sickness absence per se. If so, this parental exposure would have a lasting influence on the offspring’s norms and attitudes in an absence-prone direction, which, in turn, would act as mediators between absences in the two generations (Fig. 2). However, we cannot dismiss the alternative and non-causal explanation of confounding by factors in the parental generation, such as norms and attitudes, socioeconomic position, health, or genes [31–35]. Parental sickness absence at index person age 18 years could also be associated with parental chronic disease when the index persons were 33 years old. The associations found in the present study could therefore be confounded by participants’ burden of taking care of parents rather than a causal effect of parental sickness absence earlier in life. The distinction between a confounded and a causal relation is not trivial. If parental sickness absence has a causal effect on the next generation’s absence, measures that reduce sickness absence would have effects over generations. However, if offspring norms were influenced by parental norms, but not parental absence as such, measures aimed at reducing parental absence would have no effect on the next generation.

The exposure and outcome in this study concerned sickness absence in Norway. This health-related social benefit is dependent upon societal conditions and regulatory practices that are quite different across countries and over time [1]. Being bound to a Norwegian context, the results could be relevant for Northern Europe. Apart from this, the generalizability could be limited.

## Conclusions

Index person sickness absence was associated with parental absence 15 years earlier. The hypothesis that women would be more susceptible toward parental sickness absence was not supported by the results of this study. Studies that include data on personality traits and indicators of norms and attitudes related to sickness absence in both generations are warranted.

## Additional file

Additional file 1: Table S1. Sex- and diagnosis-specific associations between the sickness absence of parents (exposure) and index persons (outcome). **Table S2.** Sex-specific associations between musculoskeletal and psychiatric-related sickness absence of parents (exposure) and index persons (outcome). **Appendix.** Sensitivity analysis illustrating the potential impact of unmeasured confounding.
