# Supplementary material for: The gender gap in sickness absence from work and the influence of parental absence on offspring absence 15 years later: register-based cohort of Norwegians born in 1974–1976
Source: BMC Public Health. 2015 Jul 21;15:684. doi: 10.1186/s12889-015-2037-2 (PMC4509777; doi:10.1186/s12889-015-2037-2)

Table S1 Sex- and diagnosis-specific associations between the sickness absence of parents (exposure) and index persons (outcome)

Diagnostic category (index person)	Parent with absence	Women (n=38 543)			Men (n=40 335)		
		**Crude risk**	**Risk Difference[ab]**	**(95% CI)**	**Crude risk**	**Risk difference[ab]**	**(95% CI)**
All-cause sickness absence							
	Mother only	32.8	**+0.8**	**(–1.6 to +3.3)**	14.0	0	Reference
	Father only	32.3	0	Reference	17.3	**+2.3**	**(+0.5 to +4.2)**
	Neither	29.5	–2.0	(–4.2 to +0.2)	11.5	–1.9	(–2.8 to –1.0)
	Both	41.0	+8.3	(+3.8 to +12.8)	22.7	+7.5	(+4.0 to +11.0)
Musculoskeletal (ICPC L)							
	Mother only	9.0	**+0.9**	**(–0.5 to +2.4)**	6.7	0	Reference
	Father only	8.5	0	Reference	8.2	**+0.9**	**(–0.4 to +2.3)**
	Neither	6.7	–0.9	(+2.1 to –0.4)	5.1	–1.2	(–1.8 to –0.6)
	Both	14.1	+5.0	(+2.0 to +8.1)	10.9	+3.4	(+0.9 to +6.0)
Psychiatric (ICPC P)[c]							
	Mother only	7.1	**–0.0**	**(–1.4 to +1.3)**	3.6	0	Reference
	Father only	7.3	0	Reference	4.3	**+0.6**	**(–0.4 to +1.7)**
	Neither	6.0	–1.0	(–2.2 to +0.2)	2.8	–0.7	(–1.2 to –0.2)
	Both	9.9	+2.5	(–0.2 to +5.2)	5.2	+1.5	(–0.4 to +3.4)
Pregnancy-related (ICPC W)							
	Mother only	9.7	**+0.4**	**(–1.1 to +1.9)**			
	Father only	8.8	0	Reference			
	Neither	10.2	+0.7	(–0.7 to +2.0)			
	Both	7.9	–1.0	(–3.5 to +1.5)			
Other than pregnancy-related							
	Mother only	23.8	**+0.5**	**(–1.7 to +2.7)**			
	Father only	24.1	0	Reference			
	Neither	20.0	–2.6	(–4.6 to –0.6)			
	Both	33.6	+9.1	(+4.8 to +13.4)			

[a] Reference exposure is sickness absence of the opposite-sex parent; the association with sickness absence of the same-sex parent is shown in bold.
[b] In a model including mother’s and father’s sickness absence, mother’s and father’s education level, and father’s income.
[c] In order to obtain convergence, 318 men with missing information on mother’s education level were excluded from the analysis of psychiatric diagnoses.
CI: confidence interval; ICPC: International Classification of Primary Care, 2nd Edition.

Table S2 Sex-specific associations between musculoskeletal and psychiatric-related sickness absence of parents (exposure) and index persons (outcome)

Diagnostic category (index person)	Exposure (parental diagnosis)	Women (n=38 543)			Men (n=40 335)		
		Crude risk	Risk difference[a]	(95% CI)	Crude risk	Risk difference[a]	(95% CI)
Musculoskeletal (ICPC L)							
	ICPC non-L	8.4	0	Reference	7.4	0	Reference
	ICPC L	10.3	+1.6	(+0.2 to +3.0)	8.1	+0.5	(–0.8 to +1.7)
	Diagnosis missing	9.7	+1.1	(–0.5 to +2.7)	5.8	–1.7	(–2.9 to –0.4)
	No absence	6.7	–1.2	(–2.0 to –0.3)	5.1	–1.8	(–2.5 to –1.0)
Back disorder (ICPC L02, L03, L84, L86)							
	No back disorder	2.9	0	Reference	2.6	0	Reference
	Back disorder	5.6	+2.5	(+0.8 to +4.3)	2.2	–0.6	(–1.7 to +0.5)
	Missing diagnoses	2.7	–0.1	(–1.0 to +0.8)	1.8	–0.9	(–1.6 to –0.2)
	No absence	2.2	–0.4	(–0.9 to –0.0)	1.6	–0.8	(–1.2 to –0.4)
Psychiatric (ICPC P)							
	ICPC non-P	7.1	0	Reference	4.0	0	Reference
	ICPC P	10.5	+3.4	(+0.8 to +6.0)	4.8	+1.0	(–0.8 to +2.8)
	Diagnosis missing	7.1	+0.0	(–1.4 to +1.4)	3.2	–0.8	(–1.8 to +0.1)
	No absence	6.0	–0.9	(–1.6 to –0.2)	2.8	–1.0	(–1.5 to –0.5)
Depression (ICPC P03, P76)							
	No depression	3.1	0	Reference	1.5	0	Reference
	Depression	5.4	+2.3	(–0.4 to +5.1)	2.2	+0.7	(–1.0 to +2.5)
	Missing diagnoses	2.7	–0.3	(–1.2 to +0.6)	1.5	+0.0	(–0.6 to +0.6)
	No absence	2.2	–0.8	(–1.2 to –0.3)	1.1	–0.3	(–0.6 to –0.0)

[a] In a model including diagnostic-specific parental sickness absence, mother's and father's education level, and father's income. . In order to obtain convergence, analysis of male back disorders did not include mother's education level.

CI: confidence interval; ICPC: International Classification of Primary Care, 2nd Edition.

Appendix Sensitivity analysis illustrating the potential impact of unmeasured confounding

Could the adjusted risk difference estimates for offspring sickness absence in association with parental sickness absence be entirely explained by unmeasured confounding? The diagram in Figure 1 illustrates that the association between parental and offspring sickness absence, adjusted for parental education level and the father's income, still could be confounded by unmeasured parental factors (e.g., norms and attitudes, health, genes). The influence of unmeasured confounding has been assessed applying the bias formulas of VanderWeele and Arah [40].

Sensitivity analysis aimed at illustrating the quantitative impact of unmeasured confounding in realistic or worst-case examples is a tension between generality and complexity [40]. We use a simple approach because it is considerably easier to use and interpret, but it requires much stronger simplifying assumptions. In our example we assume that the set of unmeasured confounders is dichotomous and parents are either absence-prone or not absence-prone. We further assume that the confounder does not vary across strata of the covariates in the model (i.e., parental education, the father's income). Neither does the effect of the confounder on the outcome (offspring absence) vary between exposure categories (parents with and without absence).

It can then be shown [40] that the magnitude of the confounder bias d_{a+} is equal to the product of the prevalence difference δ of the confounder between exposed and unexposed and the risk difference γ of offspring absence between absence-prone and not absence-prone parents, i.e.,

$$d_{a+} = \delta\gamma$$

The observed all-cause sickness absence proportions were 0.222 for parents, 0.304 for index women, and 0.123 for index men (Table 1). In our example, we make the assumption that 30% of parents were absence-prone and 70% were not. We further assume that 40% of the absence-prone parents turned absent in the year of follow-up. That would yield an absence proportion of approximately 0.146 among the remaining 70% in order to end up with a total parental absence proportion of 0.222, similar to that observed. The prevalence difference δ is 0.400–0.146=0.254. If the confounding bias d_{a+} was equal to the adjusted risk differences between parental and offspring absence (0.034 for women (Table 3); 0.028 for men (Table 4)), the confounder–outcome association $\gamma = d_{a+}/\delta$ would be 0.034/0.254 = 0.132 for women and 0.028/0.254 = 0.112 for men. In order to yield a total female absence similar to the observed (0.304), the absence risk would have to be 0.264 for women whose parents were not absence-prone and 0.396 (0.264+0.132) for those with absence-prone parents. The corresponding absence risks for men would be 0.089 and 0.201 (0.089+0.112).

This example shows that a confounder associated with a 2.7-fold increase of parental absence and a 50% risk increase in daughters' absence could fully explain the observed association of 0.034 between parents' and daughters' sickness absence. The required confounder association with sons' absence would have to be more than doubled in order to fully explain the observed risk difference of 0.028.

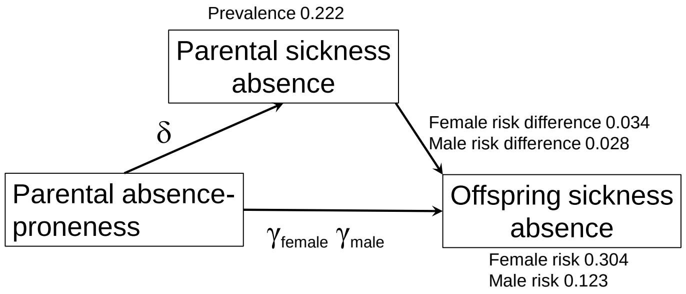

Supplement: Additional file 1: Table S1. — Sex- and diagnosis-specific associations between the sickness absence of parents (exposure) and index persons (outcome). Table S2. Sex-specific associations between musculoskeletal and psychiatric-related sickness absence of parents (exposure) and index persons (outcome). Appendix. Sensitivity analysis illustrating the potential impact of unmeasured confounding. [file 12889_2015_2037_MOESM1_ESM.pdf]
